# Cost-Effectiveness Analysis of Improving Nurses’ Education Level in the Context of In-Hospital Mortality

**DOI:** 10.3390/ijerph19020996

**Published:** 2022-01-17

**Authors:** Beata Wieczorek-Wójcik, Aleksandra Gaworska-Krzemińska, Piotr Szynkiewicz, Michał Wójcik, Monika Orzechowska, Dorota Kilańska

**Affiliations:** 1 Department of Nursing and Medical Rescue, Pomeranian University in Slupsk, Westerplatte Street 64, 76-200 Slupsk, Poland; beata.wieczorek-wojcik@apsl.edu.pl; 2 Institute of Nursing and Midwifery, Medical University of Gdansk, M. Sklodowskiej-Curie Street 3a, 80-227 Gdansk, Poland; 3 Department of Management and Logistics in Healthcare, Medical University of Lodz, Kosciuszki Street 4, 90-131 Lodz, Poland; piotr.szynkiewicz@umed.lodz.pl; 4 Rehazentrum Walenstadtberg, Chnoblisbüel 1, CH-8881 Walenstadtberg, Switzerland; michal.wojcik@kliniken-valens.ch (M.W.); monika.orzechowska@kliniken-valens.ch (M.O.); 5 Department of Coordinated Care, Medical University of Lodz, Kościuszki Street 4, 90-131 Lodz, Poland; dorota.kilanska@umed.lodz.pl

**Keywords:** cost-effectiveness analysis, education level, nurse staffing, in-hospital mortality

## Abstract

(1) Background: an assessment of the cost-effectiveness of employing an increased number of nurses with higher education from the perspective of the service provider. (2) Methods: Based on a year-long study results and data collected from a large hospital, we conducted of the costs of preventing one death. The study involved intervention by 10% increase in the percentage of nursing care hours provided by nurses with higher education. The measure of health effects was the cost of avoiding one death (CER). The cost-effectiveness analysis (CEA) was used as the evaluation method. (3) Results: The cost of employing a larger percentage of nurses with higher education amounts to a total of amounts to a USD 11,730.62 an increase of 3.02% as compared to the base costs. The estimated number of deaths that could be prevented was 44 deaths. Mortality per 1000 patient days was 9.42, mortality after intervention was 8.41. The cost of preventing one death by the 10% increase in BSN/MSN NCH percentage in non-surgical wards USD 263.92. (4) Conclusions: increasing the percentage of care hours provided by nurses with tertiary education is a cost-effective method of reducing in-hospital mortality.

## 1. Introduction

In the management of medical services, more emphasis is placed on optimising the number of personnel in healthcare facilities. The budgeting of medical services and the multitude of theoretically available possibilities of allocating funds generate the need to use economic analyses to compare various scenarios for selecting the most cost-effective interventions towards the best product for the system, in this case avoiding the endpoint of patient death. Finding the best interventions involves the use of tools and methods appropriate for the health technology assessment (HTA) as per WHO recommendations-WHA67.23 [1,2].

The concept of avoidable mortality has been evolving since the 1970s when a team of researchers led by David D. Rutsein explored a new way of examining the quality of medical care and proposed the measure of untimely deaths due to selected causes that are considered avoidable with appropriate treatment, or that could be prevented with medical care [3].

The PubMed medical database was searched for original scientific articles from 1985–2021. The search terms were: “cost-effectiveness”, “nursing”, “nurse staffing”, “in-hospital mortality”. Numerous studies were found analysing the impact of increasing the number of nursing hours or increasing the level of nursing education on in-hospital mortality, but none of them involved a cost-effect analysis. There were also studies analysing the cost-effectiveness of preventing particular adverse events in a hospital by changing the level of nursing staffing and/or education, but they did not concern in-hospital mortality. No work has been found that analyses the cost-effectiveness of an intervention, increasing the number of nursing hours per patient day (NHpPD) and/or increasing the percentage of hours of care provided by nurses with higher education on in-hospital mortality.

There is a growing body of evidence that there is a link between nursing staffing, nurse education and in-hospital mortality [4,5,6,7,8,9,10,11]. One of the most extensive studies conducted in nine European countries produced high-quality evidence on the impact of nursing hours per patient per day (NHpPD) and the level of nursing education on the incidence of adverse events, complications and in-hospital mortality. The above results indicate the need for similar multidimensional analyses, especially in the countries which have not been covered by such studies yet [12].

There is also published research which does not yield conclusive results. Twigg’s systematic review of investment in improving nursing staffing did not identify conclusive evidence of the cost-effectiveness of this investment in improving patient safety from the cost–benefit perspective [13,14]. Hence, there is still a need for further research and evidence regarding the importance of nurses’ education level.

In 2018, a system of gradual remuneration of nurses was introduced in Poland. It is education-centred, which means that it is based on the coefficient assigned to education level [15,16,17]. Consequently, as of 1 January 2019, new employment standards apply to nurses in Polish hospitals. In non-surgical wards, there are 0.6 full-time job positions per bed, and in surgical wards, the value is 0.7. However, there are no national recommendations for the optimal nursing staffing ratio or skill mix, which would help nursing managers manage staff to minimise the risk of adverse events.

In Poland, there are two levels of nursing education: nurses with higher education—bachelor’s degree nurse in nursing (European Qualification Framework, EQF level 6, BSN) and master’s degree nurse (EQF level 7, MSN) and a lower level-certified nurses who graduated from nursing high schools (EQF level 4/5). These schools were in operation until 2002 [18].

Due to the insufficient number of nurses and one of Europe’s lowest rates of employed nurses per 1000 inhabitants, the debate on the restoration of vocational schools for certified nurses has been resumed in Poland. Such discussions are also taking place in other countries with low numbers of nurses. Consequently, the need has arisen to assess the clinical effectiveness and cost-effectiveness of increasing the level of nurse education, particularly in terms of patient safety and the cost of such an investment from the perspective of service providers, payers and the public. In the last decade, the overall in-hospital mortality slightly decreased in Poland from 2.3% in 2010 to 2.0% in 2018, which may be a measure of the quality of treatment [19]. More and more nurses have higher education: 16% in 2013 and 30% in 2020 [20]. According to the Polish Accreditation Committee (Centre for Quality Monitoring in Health Care, CMJ), the analysis of in-hospital mortality and avoidable mortality may be a valuable tool for assessing and improving the level of health care.

This study aims to assess the cost-effectiveness of employing an increased number of nurses with higher education from the perspective of the service provider.

## 2. Materials and Methods

There are no studies exploring the impact of nursing staffing on adverse event rates and the costs of these investments in Poland. The nursing staffing level in Poland was assessed based on a study carried out over three years, from January 2012 to December 2014, in a large (523 beds) specialised hospital. It was a case-control study with a retrospective analysis of the medical records of hospitalised patients and personnel records of the nursing staff. The research showed that a higher percentage of nursing hours provided by BSNs/MSNs is associated with a lower number of deaths in non-surgical wards. A 10% increase in NCH percentage provided by BSNs/MSNs (BSN/MSN NCH) reduced the mortality rate by 7.53 per 1.000 patient-days [10].

The cost-effectiveness analysis (CEA) was used as the evaluation method. We have used economic model methodology-CEA analysis which was used in the NICE (National Institut for Health Excellence) Report by Cookson [21].

The economic model was populated with the effectiveness results from a Polish study by Wieczorek–Wojcik. It assessed the hospital standard care as the percentage of nurses with higher education in non-surgical wards [10]. The year 2014 was used for the CEA analysis due to the fact that in the years 2012–2014, the percentage of NHPPD provided by nurses with higher education gradually increased (24.62 ± 16.78 to 39.16 ± 17.82).

The study population consisted of adults hospitalised in 4 non-surgical wards. In the analysed period 6031 patients were hospitalised, which amounted to 43,502 patient-days. The mean age of the hospitalised patients was 57.4 years. The 410 deaths in not-surgical patients were included in the analysis. The focus of the study was on the percentage of hours of nurses with higher education in non-surgical departments (32.2%). The intervention was to increase the percentage of hours of nurses with higher education by 10% (to 42.2%).

Patients received an intervention which consisted in increasing number of nursing care hours (NCH) provided by BSNs by 10%. The number of additional hours of nursing care provided by BSNs/MSNs was calculated by multiplying the number of BSN/MSN NCH in individual wards by 10%.

The cost of the intervention was calculated by subtracting the cost of one hour of care provided by a nurse without higher education from the cost of one hour of care provided by a nurse with higher education and multiplying the result by the number of additional BSN/MSN NCH The cost of one nursing hour was calculated by dividing the total salary of nurses by the actual number of NCH in the analysed period. The cost of one hour of care provided by a nurse with higher education was calculated by dividing the total salary of BSNs/MSNs by the actual number of BSN/MSN NCH. The costs of one hour of care provided by a nurse without higher education were correspondingly calculated.

The number of deaths was estimated in the following non-surgical wards: general wards, pulmonology, neurology, and cardiology. We calculated the mortality rate per 1000 patient days before and after the intervention consisting in adding 10% more nursing hours provided by nurses with higher education. The difference in mortality between the interventions (incremental change) was then estimated. In the next step, we calculated the number of deaths that could be avoided by increasing the number of nursing hours provided by nurses with higher education by 10%. Avoidable deaths were calculated for individual wards by dividing the number of additional nursing hours provided by nurses with higher educated by 7.53 per 1000 patient-days.

CER (cost-effectiveness ratio) is the measure of the results of the intervention profitability analysis which was used to calculate the cost of avoiding one death. It is the basic indicator for assessing the results of the compared alternatives as the cost of the effect unit. This indicator was compared to the revenue gained by the service provider per one patient.

The Bioethics Committee of the Medical University of Gdansk approved the presented research.

## 3. Results

**Figure 1:**
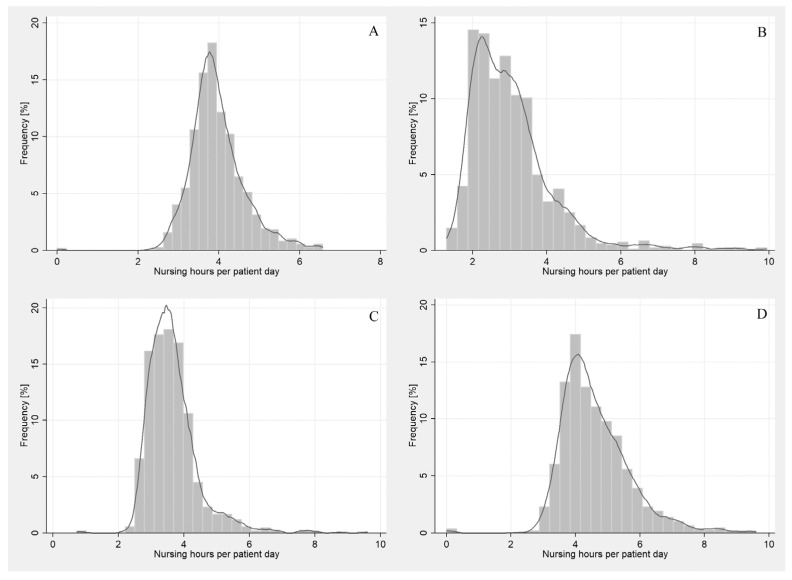
Histogram and data distribution model for the nursing care index (NHPpD) in non-surgical wards: (**A**) general ward, (**B**) pulmonology, (**C**) cardiology, (**D**) neurology.

The distribution of the level of nursing hours per patient per day (NHPpD) in non-surgical wards shows the ranges for the analysed hours of nursing care and their frequency for the following four wards—Figure 1.

The percentage of nursing hours provided by nurses with higher education in non-surgical wards was 32.2% on average, with the highest value in the neurology ward (46.3%) and the lowest-in the pulmonology ward (19.2%)—Table 1.

Backward stepwise regression (β (SE)/100) confirms the impact of nurses’ education (HE—higher education) on frequency in-hospital mortality (–0.7529 (0.1385) ^USD^). The variables for which the significance level p was lower than 0.001 were considered statistically significant parameters. It is clear that a sensible skill-mix intervention would be a 10-percentage point increase in the proportion of registered nurses (HCAs) from a ward average of 32.2% to 42.2%.

The total number of nursing hours in non-surgical wards was 177,723. For the analysed wards, a 10% increase in the percentage of BSN/MSN NCH corresponded to the total number of 5905.2 h (Table 2).

The estimated cost of the intervention was calculated based on actual data on NCH. In the analysed departments, the total salary of nurses in non-surgical wards was USD 784,799.66; the salary of nurses BSNs/MSNs amounted to USD 381,370.21. The average revenue per patient in the analysed year was USD 1096.10, and the average cost per patient was USD 1038.91 (Table 3).

The average cost of one nursing hour was USD 4.45 in non-surgical wards. The average cost the cost of an hour of care provided by BSNs/MSNs was USD 6.56. The cost of employing a larger percentage of BSNs/MSNs amounts to USD 11,730.62 an increase of 3.02% as compared to the base costs, i.e., the actual cost of remuneration of nurses without higher education. These amounts were calculated by deducting the remuneration of nurses without higher education from the cost of employing an additional percentage (10%) of BSNs/MSNs. The cost of increasing one hour the percentage of nurses with higher education by 10% per patient amounted to USD 1.95. In order to for the payer to finance these analysed interventions, the income per patient in non-surgical wards should increase by 0.2%.

The estimated number of deaths that could be prevented by adding an extra 10% increase in the BSN/MSN NCH percentage in non-surgical wards was 44 deaths. The total number of deaths before the year-long intervention was 410. The number of deaths in the general ward was 267, pulmonology—34, neurology—60, cardiology—29. The number of deaths that could be avoided in general ward was 17, pulmonology—5, neurology—15, and cardiology—8.

Mortality per 1000 patients a day was 9.42, mortality after the intervention was 8.41. This means reducing in-hospital mortality by 1.01 deaths per 1.000 patient-days in non-surgical wards is the incremental change.

### 3.1. Cost-Effectiveness Analyses

The cost-effect ratio (CER) was calculated by dividing the estimated cost of this intervention by the number of avoidable deaths. The cost of preventing one death by the 10% increase in BSN/MSN NCH percentage in non-surgical wards was USD 263.92 (Table 4).

#### Sensitivity Analysis

In order to obtain the most realistic results, a sensitivity analysis was performed. This method uses extreme but possible variable values that may affect the result, i.e., the cost of avoiding one death. By simulating the optimistic and the pessimistic scenario, we obtained objective results reflecting the possible outcomes of a given intervention in the future. The sensitivity analysis shows that the results in both extreme cases do not differ significantly, which indicates a small risk of obtaining an intervention result different from the one that the economic analysis indicated.

The adopted sensitivity analysis parameters for the 10% increase in the percentage of nurses with higher education include the number of patient-days and the cost of nursing care hours. The parameters and calculations used in the sensitivity analysis:

1. Number of nursing hours with BSN/MSc—the standard deviation was calculated from quarterly data selected in the intervention in the wards; the results was multiplied ×4 quarters and subtracted from the base number of nursing hours (pessimistic scenario) or added to the base number of nursing hours (optimistic scenario).
2. Cost of one nursing hour with BSN/MSc—we calculated the standard deviation from the annual data for the medical treatment ward and added to the base cost (pessimistic scenario).
3. Number of avoidable deaths—we calculated the standard deviation from the annual data for the medical treatment ward and added to the base number of deaths (pessimistic scenario) or subtracted from the number of deaths (optimistic scenario).

A deterministic multi-directional sensitivity analysis was performed. To that end, we selected variables with a high degree of uncertainty/variability in the analysed area (Table 5).

In the sensitivity analysis, the optimistic and pessimistic scenarios were simulated for the intervention consisting in increasing the percentage of BSN/MSN NCH by 10% in non-surgical wards. The purpose was to assess how much the planned intervention is at risk of a result inconsistent with that predicted in the economic analysis.

In the conducted sensitivity analysis for the 10% increase in the BSN/MSN NCH intervention percentage in non-surgical wards, in the optimistic scenario, a decrease in the number of nurses hours to 4899.24 will consequently decrease CER to USD 233.82. In the pessimistic scenario, an increase in the number of 6906.16 nursing hours will result in a CER increase to USD 329.60. That means that such a change in cost makes the intervention unprofitable for the hospital with the current financing by the payer (USD −65.7).

In the optimistic scenario, according to the adopted assumptions, if the number of avoidable deaths is increased by the standard deviation 49.6, CER will decrease to USD 236.66. In the pessimistic scenario, if the number of deaths decreases to 39.3, CER will increase to USD 298.28 which means that such a change in cost makes the intervention unprofitable for the hospital with the current financing by the payer (USD −34.36).

According to the adopted assumptions, if the cost of an hour of nursing care provided by nurses with higher education increases by the standard deviation, CER increases to USD 408.67, which means that such a change in cost makes the intervention unprofitable for the hospital, with the current financing by the payer (USD −145). The optimistic scenario does not assume a decrease in the cost of an hour of care, so the variable has no impact on CER.

In order to assess the cost-effectiveness of the increase in the number of hours of nurses with higher education from the social perspective, CBA (cost-benefit analysis) was used. This analysis showed a gross monetary benefit to society of USD 14,790,547.93. That involves avoiding the deaths of 44 patients. In 2014, the World Bank estimated the value of a statistical life (VSL) in Poland at USD 332,765.9. This publication calculates how much has to be spent in PLN to save 1 PLN. The analysis was performed with the use of CBR (cost/benefit ratio) and the result was obtained in PLN and then converted into USD. The result of the analysis shows that each PLN saved by increasing the number of hours of care provided by nurses with higher education is associated with the cost of USD 0.0002 (PLN 0.0008). The BCR benefit/cost ratio is USD 359.57 (PLN 1.261), which means that each PLN invested in increasing the number of hours of nursing care provided by nurses with higher education returns USD 359.57 (PLN 1.261) in benefits.

The change in the cost of avoiding one death (CER) depending on the scenario is presented in Table 5.

## 4. Discussion

This is the first Polish study to analyse the cost-effectiveness of increasing the level of nurse education in the context of in-hospital mortality. The cost of avoiding one death (CER) with the intervention consisting in increasing in the percentage of BSN/MSN NCH in non-surgical wards by 10%, the CER is USD 263.92.

In the CEA economic analysis conducted for the purposes of this article, the first step involved answering the question what interventions will be compared and determining the cost-effectiveness perspective from which the assessment is conducted. The selected study population included adult patients of non-surgical wards. In the temporal perspective, the studied period was 2014. The intervention involved increasing the number of hours of nurses with higher education by 10% in relation to the comparator, i.e., the standard level of hours of care for nurses with higher education, which amounted to 32.2%. It resulted from a previously conducted case-control study. It was assumed that the health effect would be a reduction in the number of deaths. The cost-effectiveness analysis was carried out from the perspective of the service provider. The results were then specified, and the intervention costs estimated. The number of additional hours of care for nurses with higher education was calculated and valued. An Excel spreadsheet was used for the analysis. Unit cost per hour of nursing care was calculated, cost per an hour of care provided by a nurse with higher education and the total cost was calculated. The costs of nursing hours were calculated on the basis of data obtained from the SGA Report for 2014 [22]. The analysis included deaths in non-surgical wards which occurred in 2014. Then, the process of extrapolating the intermediate results, which are deaths to end results, i.e., avoidable deaths. The number of avoidable deaths was calculated. The ratio of 7.35 per 1000 patient days was used to calculate avoidable deaths. It was obtained in a previously conducted case-control study [10]. In the next step, the cost per unit of the effect was calculated—avoidable death CER. The result of the analysis indicates that in order to make a decision to reject or accept the intervention, the service provider should independently determine the level of CER that is acceptable. Hence, they may need additional information. On the other hand, in 2013, eleven member states, including Poland received a recommendation to reform their healthcare systems within the European Semester. Most of these recommendations focused on sustainability and cost rationalisation in health systems, calling for reforms in the hospital sector and in pricing health care services [23].

The costs of the described intervention consisting in increasing the number of hours of care provided by nurses with higher education by 10% turned out to be high, but very effective compared to the comparator. The cost of USD 1.95 for each hospitalised patient, according to the authors, appears to be an acceptable cost for the healthcare provider, especially now that a record low number of nurses is reported in Poland. In 2020, the number of nurses per 1000 citizens was 6 nurses [24].

According to the authors, the shortage of staff will force the entities managing the entities to accept additional costs. In health care, market prices do not always reflect the real value of a given resource. This also applies to the salaries of nurses. The change in the costs of nurses’ salaries may be the result of pressure from the professional group and not the market value of the nurses’ qualifications. The deficit of staff in the world market, numerous strikes related to the financial demands of nurses may force hospitals to ensure the continuity of work and increase salaries above the cost-effectiveness level.

In the sensitivity analysis, the CER result indicators were calculated when the parameters of some estimated data were changed. The aim was to check the reliability and credibility of the results. The calculations were made in an Excel spreadsheet. Two scenarios of data volatility were adopted: pessimistic and optimistic. In the sensitivity analysis model, values were adopted for the variables that affect the final effect, i.e., the number of hours of nursing care provided by nurses with higher education, the cost of one hour of nursing care provided by a nurse with higher education, and the number of avoidable deaths. The analysis carried out for the purposes of the publication indicates that the changing cost of an hour of work of a nurse with higher education is a greater cost than the changing number of hours. In the scenarios where we deal with the variability of the described parameters, the intervention generates costs which, in the opinion of the authors, the service provider may be able to incur. In view of the above, the results of the analysis are the basis for a recommendation to continue employing nurses with higher education in non-surgical wards.

A similar study in the UK estimated that the number of deaths avoidable through increasing the average number of NHPPDs by one hour corresponded to an 8% reduction in the risk of death. The study indicated that increasing the number of NHPPD by one hour involves greater benefits with greater costs. The intervention cost was estimated at GBP 10.1 million annually, with a gross cost per patient GBP 219 and cost of life saved GBP 69.097. Increasing employment reduces the risk of death by 2% and costs GBP 70 per patient. Assuming an increase in staff structure, a life saved is valued at GBP 7.015. On a pessimistic basis, the estimated life saved value is GBP 21.013. An increase in the staff structure is an estimated gross cost of GBP 1.3 million annually (0.3% of budget); GBP 26.351 for one saved life and GBP 28 per patient [25].

Shamliyan’s research has shown that the greatest economic benefit from improving nursing staffing corresponds to an increase of 0.56–1.5 in full-time positions (FTE) for higher education nurses per patient-day, and the hospital’s cost of remuneration exceeds the benefits [9]. According to Rothberg, the cost of one saved life is USD 56.394 with a reduction in patients per nurse from 8 to 7, and USD 174.464 with a change from 5 to 4 patients [26]. The study by Behner et al. indicated that a 20% drop in the employment of nurses meant the costs of complications were higher by USD 28.441, which exceeded the savings resulting from redundancies [27]. In a study by Weiss et al., it was shown that the payer saves USD 652 for hospitalisation, but the hospital loses USD 213 per patient when the number of care hours provided by BSNs/MSNs was higher by one standard deviation, i.e., 0.75 NHpPD. The cost of this intervention per patient was USD 198, and the payer saving was USD 607 per patient [28].

Verification of the clinical effectiveness and cost of the impact of qualified nurses (nurse practitioners) on the clinical outcomes of patients hospitalised in surgical and non-surgical wards indicated a link with lower in-hospital mortality, but these clinical benefits are associated with higher financial costs. However, the improvement in nursing care in non-surgical wards is all the more important, because, according to research, the risk of death for these wards is more than 50% higher, and the probability of avoidable death is lower than in surgical wards [29].

The results indicate that while higher education influences the cost of nursing hour care in Poland, the extent of that influence is small compared to that exerted by work experience. At the same time, there is a strong correlation between the percentage of BSNs/MSNs and the reduction in the number of deaths in wards. This indicates higher effectiveness with BSNs/MSNs. Given the above results, the higher-level nurse education and training reform initiated in the 1990s in Poland were justified. As a consequence, the percentage of BSNs/MSNs increases [20]. It can be thus concluded that the effectiveness of nursing care will also increase. However, the relationship between the increase in the percentage of BSNs/MSNs and the number of deaths should be monitored. Hence, it is justified to recommend further research in this area.

Hospitals in Poland are indebted, and their financial situation is difficult. On the other hand, one of the dimensions of Triple Aim, a framework developed by the Institute for Healthcare Improvement (IHI) to describe an approach to optimising the performance of a healthcare system, is cost reduction [30]. Therefore, managers of medical entities are trying to minimise treatment costs. The European Commission discusses the appropriate costing of health services [31]. Thomas et al. have identified three resilience types-financial, adaptive and transformational [32,33]. The system of financing health services in Poland is not based on the dependence of financing on the effects in line with the VBHC methodology. Such a system would make it possible to finance the effectiveness of nurses’ work in the context of clinical outcomes, e.g., in-hospital mortality. This approach has been thought to be only beneficial from a social or systemic perspective rather than a single hospital. The presented study shows that it will also be beneficial for the hospital’s financial performance. This is also confirmed by the latest analysis by Griffiths et al., who found that flexible and resilient nurse staffing options contribute to cost-effective hospital care and address staff shortages [34].

When analysing the possible benefits for the payer, it is worth adopting the perspective of social benefits, which are analysed in HB HTA. This may indicate that it is more profitable to invest in the education of nurses than to increase the NHpPD number. The intervention consisting in a 10% increase in the percentage of BSN/MSN NCH in non-surgical wards is associated with avoiding the 1.01 avoidable deaths per 1000 patients a day. The relatively low cost-effect ratio characterises this intervention, and its introduction is a benefit for the hospital.

So far, the assessment of the effectiveness of the employment of nurses has mainly been based on CEA and CUA economic analyses regarding the service provider without considering the social perspective [35,36,37]. The methodology recommended by the WHO for estimating the costs of eliminating negative treatment results, including deaths, is the Hospital-Based Health Technology Assessment (HB HTA) methodology [1]. HB-HTA serves to increase the possibilities of managing the health care system at the local level (hospital directors) and (indirectly) at the national level (EU) [13,14].

In nursing practice, conducting case–cost analyses is difficult due to the lack of cost data and the challenges and time needed to obtain them. Additionally, in Poland there are no central databases enabling statistical analyses that can be used to measure health events and their costs. Such resources would enable economic analysis to be carried out on a larger scale. It is true that clinical data are becoming easier to obtain due to the functioning of hospital information systems (HIS). Nevertheless, the quality of clinical data and their incompleteness often complicate conducting studies. Economic analyses require identifying the costs incurred to cover the employment of nurses as well as social benefits of the interventions undertaken. This is important for evidence-based health policy that contributes to building the resilience of health systems. A case in point, EU Member States are recommended to invest in developing their information flows to, for example, ensure that patient-level information flows are channelled appropriately to all necessary healthcare providers, or that more effective and sustainable reorganisation of health systems and services are supported. The report of the eHealth Task Force entitled “Redesigning Health in Europe for 2020” urges decision-makers to use the power of data [38].

The pandemic situation showed that the change in the patient’s morbidity profile may undoubtedly have a significant impact on the effectiveness of interventions undertaken by nurses in the context of avoidable deaths.

Another important perspective raised by the authors is the assessment of the profitability of reducing hospital mortality in the social perspective based on data from World Bank experts [39], which is still limited.

In recent years, Poland has seen a dynamic growth of interest in using economic analyses to make rational allocation decisions in the health sector and ensuring transparency in spending public funds. The Agency for Health Technology Assessment and Tariffs (AOTMiT) rates medical interventions, including nursing interventions [40]. Economic analysis is the most important practical contribution that economics has made to the decision-making process in health care [41]. This approach was adopted by the European Commission in 2014, which indicated that Health Technology Assessment (HTA) is the main way to provide a common method for assessing the effectiveness of interventions and proper costing of services, thus enabling decision-makers to allocate resources as efficiently as possible. The ability to calculate healthcare services accurately is not only necessary for controlling expenditure, but also a prerequisite for effective decisions on investment and prioritisation [23].

The additional costs of changing mortality should be paid for by increasing the health premium in relation to GDP. The health premium in Poland is relatively low compared to other countries and amounts to 9% [42]. There are ongoing discussions about the planned regulations in this area, as it is undoubtedly necessary to gradually increase it, following the example of other countries. A higher standard of care could also be possible under additional insurance, which is not available in Poland. Currently, also in Polish hospitals, thanks to the development of HB HTA carried out by AOTMiT, the cost accounting standard is being implemented. It will undoubtedly allow the monitoring of the actual human resources and, possibly, the effectiveness of care. Ultimately, it will contribute to savings that can be used to cover the costs of additional employment of qualified nurses, as shown by many studies [12], as well as own research [10]. In Poland, hospitals settle accounts with the payer by coding benefits using diagnosis-related points according to the ICD-10 classification. There is no classification that would allow for the accounting of nursing services. Implementation of the classification dedicated to nursing services, such as ICNP, would make it possible to estimate the cost of nursing services. Ultimately, it would allow the payer to separate the financing of nursing services. It would also provide financing for the employment of nurses with higher qualifications (for instance, higher education) in the hospital.

In line with the methodology used in studies of avoidable mortality in developed countries after 2000, the upper age limit was 75 years [43,44]. In our study, the mean age of the patients was 57.4 years.

The endpoint of the presented study is death. Death in the elderly age (after age 70) is highly probable, but death in middle age (30–69 years) may not be common [45]. It is estimated that of all deaths worldwide in 2010, 57% were avoidable. More than 27% of all deaths from chronic diseases were in those under the age of 60 and were avoidable [46]. In 2012, as much as 68% of all deaths in the world (38 million out of 56 million deaths worldwide) occurred due to chronic non-communicable diseases. According to WHO, chronic non-communicable diseases were responsible for 90% of deaths in 2014 in Poland [47].

It should be noted that when staff without higher education is referred to in Poland in this context, it means nurses who graduated from medical secondary schools. In Poland, such secondary schools stopped enrolling new students in 1991, which means that the last nurses who graduated from those schools will be in the system for at least 9 years before they retire. In addition, the system at that time made it possible to obtain a license without a taking the final high school examination, which is otherwise a mandatory condition for applying to a university. Then, in 2005, special ‘bridging’ college programmes were designed that were dedicated for nurses who could thus complete their education and obtain a bachelor’s degree [48]. Almost 50,000 nurses completed those programmes. Initially, the nurses who enrolled had to pay tuition. Then, from 2008 to 2015 those programmes were offered as part of an EU project, and they were free of charge for students [49].

Some nurses could not undertake the bridging programmes as they had not taken and passed the final secondary school examination. As of 2005 in Poland, nurses are educated only as part of higher education at accredited universities. Due to the insufficient number of nurses on the labour market, politicians are revisiting the idea of restoring nursing education in medical secondary schools [50].

Evidence suggests that increasing nursing staffing and/or changing the required educational attainment have a positive effect on patient outcomes, but this effect comes at a cost [10,12]. It is up to the payer to determine whether this cost is acceptable or not. It may be unprofitable from the hospital’s perspective but profitable from the social perspective because upgrading the qualifications of nurses can be more cost-effective than additional NHPPD.

## 5. Conclusions

Analysis CEA in four non-surgical wards showed that increasing the percentage of nurses with tertiary education is a cost-effective method of reducing in-hospital mortality. An investment in financing nursing care is socially beneficial and may also be beneficial for the payer. Employing nurses with higher education in a hospital is a profitable investment that leads to reducing avoidable mortality. It is recommended that skill mix should be implemented in hospitals and competencies should be organised. The issues presented in this paper should be further explored in a broader multicentre perspective based on the HB HTA methodology, because the obtained CEA score indicates that a healthcare provider needs additional information to make a decision to reject or accept an intervention. It would be helpful if there was a defined level of CER that an intervention must achieve in order to be considered cost-effective and, consequently, acceptable for implementation.

### Limitations

This study is based on data collected on patients in one healthcare entity. In order to obtain a complete and credible picture, further studies are needed to cover a larger group of subjects and a higher number of medical entities. Moreover, the study adopts the employer’s perspective-in this case, the hospital-when assessing the cost–benefit perspective.

The paper does not consider the social costs incurred by the payer, e.g., training nurses; the social benefits resulting from avoiding one death. From the point of view of an individual hospital, it is possible to increase the number of employees or increase the percentage of care hours provided by BSNs/MSNs. However, in the country’s perspective, it may pose a threat to other entities due to potential market drainage.

The assumptions for the sensitivity analysis included the same changes in the cost of an hour of nursing care with and without higher education. This scenario was considered the most likely. However, cost changes will likely occur in only one of the groups of nurses mentioned above.

The study indicates that the percentage of nurses with a university degree varies significantly between wards. With an overall increase by 10%, the lowest increase is noted for the wards with the lowest number of nurses. This is a limitation and a problem that affects drawing conclusions.

## Figures and Tables

**Table 1:** Values of descriptive statistics for the percentage of nurses with university education over the entire period of observation in individual wards, broken down by non-surgical wards.

Wards	M	Me	Min	Max	Q_1_	Q_3_	SD
General Ward	38.4%	36.4%	0.0%	83.3%	30.0%	45.5%	12.5%
Pulmonology	19.2%	16.7%	0.0%	75.0%	12.5%	28.6%	14.6%
Cardiology	24.2%	25.0%	0.0%	60.0%	12.5%	33.3%	12.3%
Neurology	46.8%	45.5%	0.0%	90.9%	37.5%	55.6%	14.7%
Non-surgical wards	32.2%	33.3%	0.0%	90.9%	20.0%	44.4%	17.5%

Legend: min, minimal; max, maximal; M, mean; SD, standard deviation, Q1, lower quartile; Q3, upper quartile.

**Table 2:** Nursing care hours (NHC) before and after intervention.

Wards	Total Number of Nursing Hours	Number of NHC Provided by Nurses with Higher Education	10% Increase in the Number (Percentage) of NHC Provided by Nurses with Higher Education
General ward	57,330	22,072	2207.2
Pulmonology	32,487	6205	620.5
Neurology	42,042	19,675	1967.6
Cardiology	45,864	11,099	1109.9
Non-surgical wards TOTAL	177,723	59,051.8	5905.2

**Table 3:** Revenue and cost per patient by non-surgical wards.

Wards	Gross Salary of Nurses (USD)	Gross Salary of Nurses with Higher Education (USD)	Number of Patient Days	Number of Patients of the Year	Total Costs (USD)	Average Cost per Patient (USD)	Average Revenue per Patient (USD)
General Ward	262,736.84	171,831.47	16,835.00	2073.00	1,972,564.39	951.29	1172.49
Pulmonology	116,934.49	41,183.65	7201.00	1129.00	924,412.09	818.59	948.23
Neurology	198,297.31	97,696.53	8797.00	1028.00	1,493,281.08	1450.17	1485.56
Cardiology	206,831.02	70,658.57	10,669.00	1801.00	1,687,290.03	935.61	878.57
Non-surgical wards TOTAL	784,799.66	381,370.21	43,502.00	6031.00	6,077,547.59	1038.91	1096.10

**Table 4:** The cost of additional interventions per patient and the cost-effect ratio (CER) of avoidable deaths.

Wards	The Cost of Increasing the Percentage of Nurses with Higher Education by 10% (USD)	The Cost of Increasing the Percentage of Nurses with Higher Education by 10% per Patient (USD)	The Number of Avoidable Deaths	CER: Increasing the Percentage of Nurses with Higher Education by 10% (USD)
General Ward	7225.54	12.22	17	435.87
Pulmonology	1907.94	1.69	5	406.22
Neurology	498.41	0.48	15	33.64
Cardiology	2098.74	1.17	8	251.12
Non-surgical wards TOTAL	11,730.62	1.95	44	263.92

**Table 5:** Sensitivity analysis.

	CER—Avoidable Deaths		
	Increasing the Percentage of Hours of Care Provided by Nurses with Higher Education by 10% in Non-Surgical Wards		
	Variant		
	Standard	Pessimistic	Optimistic
The number of nursing hours with BSN/MSc (PLN)	925.57	1155.92	820.01
The number of nursing hours with BSN/MSc (USD)	263.92	329.60	233.82
The cost of one nursing hour with BSN/MSc (PLN)	925.57	1432.85	925.57
The cost of one nursing hour with BSN/MSc (USD)	263.92	408.57	263.92
The number of avoidable deaths (PLN)	925.57	1 046.07	829.97
The number of avoidable deaths (USD)	263.92	298.28	236.66

## Data Availability

The patients and nurses’ data cannot be shared by the investigators under the data use agreement with the hospital in Wejherowo; however, the original data collection can be requested directly from the hospital in Wejherowo.
